# A brief history and spectroscopic analysis of soy isoflavones

**DOI:** 10.1007/s10068-020-00815-6

**Published:** 2020-09-15

**Authors:** Young Sung Jung, Chan-Su Rha, Moo-Yeol Baik, Nam-In Baek, Dae-Ok Kim

**Affiliations:** 1 Department of Food Science and Biotechnology, Kyung Hee University, Yongin, 17104 Republic of Korea; 2 Department of Oriental Medicinal Biotechnology, Kyung Hee University, Yongin, 17104 Republic of Korea

**Keywords:** *Glycine max* (L.) Merrill, High-performance liquid chromatography, Mass spectrometry, Nuclear magnetic resonance, Ultraviolet spectrum

## Abstract

The production of soybean continues to increase worldwide. People are showing more interest in the beneficial health effects of soybeans than before. However, the origin and history of soybeans are still being discussed among many researchers. Chromatographic methods enable the desirable separation of a variety of isoflavones from soybeans. The structures of isolated soy isoflavones have been successfully identified in tandem with spectroscopic analytical instruments and technologies such as liquid chromatography, mass spectrometry, and nuclear magnetic resonance spectroscopy. The theoretical background behind spectroscopy may help improve the understanding for the analysis of isoflavones in soybeans and soy-derived foods. This review covers the origin of the English name of soybean and its scientific name, *Glycine max* (L.) Merrill, based on the evidence reported to date. Moreover, the reports of soy isoflavones discovered over a period of about 100 years have been briefly reviewed.

## Introduction

Soybeans (*Glycine max* (L.) Merrill) are among the six most produced crop in the world, and their processed products are widely used for human diet and animal feed. World soybean production in 2018/2019 was estimated to reach 398 million tons (Carneiro et al., 2020; Kofsky et al., 2018). Soybeans are the main source of isoflavones, a subclass of polyphenols with a high added value. The biological activity of isoflavones has been well reported. Isoflavones, known as phytoestrogens, are biologically active compounds with weak estrogen activity (Zaheer and Akhtar, 2017). Isoflavones are known to lower the incidence of steroid hormone-dependent cancers such as breast, prostate, and colon cancer (Bustamante-Rangel et al., 2018). Also, isoflavones have been proven to help prevent and treat several dysfunctions and diseases related to aging, including neurodegenerative disorders, osteoporosis, metabolic and cardiovascular diseases, and symptoms of menopause (Bustamante-Rangel et al., 2018; Jayachandran and Xu, 2019). In order to evaluate the various health benefits of isoflavones, however, accurate measurements of individual isoflavone contents, total isoflavone content, and the half-maximal inhibitory concentration (IC_50_) are necessary. It is the same when one attempts to study the expected biological activity from isoflavones through the ingestion of soybeans and soy-derived foods.

First, we looked at the origins of soybeans and the history of the identification of isoflavones in chronological order. Literature referring to the history of soybeans may provide an opportunity to identify the origin of soybeans more clearly. Secondly, we looked at the emergence and development of spectroscopic instruments that have served as the cornerstone of soy isoflavone discoveries. The analysis methods of liquid chromatography (LC), mass spectrometry (MS), and nuclear magnetic resonance (NMR) and their results can increase the understanding of soy isoflavones.

## History and origin of the English name and scientific term for soybeans

The history of soybeans has been well organized by researchers (Hymowitz, 1970; Martin and Leonard, 1949; Pratap et al., 2012). When relating to the origin of soybeans, the phrases “one of the oldest cultivated crops” and “known for over 5000 years” have been repeatedly used for more than half a century (Hymowitz, 1970; Martin and Leonard, 1949). Hymowitz (1970) mentioned that these descriptions about soybeans have repeatedly appeared from one publication to another in agronomic fields without any citation or explanation. The facts that are now being clearly and commonly discussed are where (northeast of China) and when (1500–221 B.C.) soybeans first emerged (Hymowitz, 1970). Ancient pictograms of soybeans in bronze inscriptions were proposed to have appeared in approximately the eleventh century B.C. (Shurtleff & Aoyagi, 2014), which is the period of Shang (ca. 1500–1027 B.C.) or Chou (ca. 1027–221 B.C.), revealing that soybeans were domesticated during these ancient Chinese dynasties (Shurtleff and Aoyagi, 2014). As the dynasty regime expanded and the trading volume increased, the soybean was introduced to South China, Korea, Japan, and Southeast Asia (Hymowitz, 1970). The first record of soybeans was found in the Chinese encyclopedia “*Pên Ts’ao Kong Mu*”, written in 2838 B.C. (Shurtleff and Aoyagi, 2013). To date, it has been speculated that soybeans emerged during the Shang Dynasty or even earlier, but researchers’ discussions on its origin continue.

Soybean belongs to the family Fabaceae (or Leguminosae), and its scientific name *Glycine* was originally coined by the “father of modern taxonomy”, Carl von Linné (Hymowitz and Newell, 1981; Linné, 1754). The word *Glycine* is derived from the Greek word *glykys*, which means sweet. Originally, however, the *Glycine* genus introduced by Carl von Linné did not refer to any of the current *Glycine* species. The first taxonomic names for cultivated soybeans, *Phaseolus max* and *Dolichos soja*, were described by Linné in 1753 (Hymowitz and Newell, 1981). Later, the scientific name and classification of soybean was established under the international botanical rules (Ferraz de Toledo et al., 1994). The genus *Glycine* Willd. is made up of two subgenera, *Glycine* (perennials) and *Soja* (Moench) F.F. Herm. (annuals) (Hymowitz, 2004; Hymowitz and Newell, 1981; Pratap et al., 2012). *Glycine max* (L.) Merrill and *G. soja* Siebold & Zuccarini belong to the annual species (Hymowitz, 2004; Pratap et al., 2012). *Glycine max* indicates domesticated soybeans, while *G. soja* denotes wild soybeans (Li et al., 2010). The species name *G. max* (L.) Merrill, where the L. stands for Linné, was derived from Elmer Drew Merrill in 1917 (Hymowitz and Newell, 1981; Pratap et al., 2012). The name *G. soja* Siebold & Zuccarini, a progenitor of the cultivated soybean described by Siebold and Zuccarini in 1846 as a new species, was proposed by Bernard Verdcourt in 1970 (Hymowitz and Newell, 1981; Verdcourt, 1970).

There have been many discussions about the origin and etymology of the English name of soybeans. The most definite theory based on a reference (King, 1830) is that the English name of soybean is derived from the Japanese soy sauce. In 1679, philosopher John Locke, first mentioned “soy sauce” in English in his journal published in 1829 (King, 1830). The soy sauce made in Japan was probably exported by Dutch merchants (Shurtleff and Aoyagi, 2014). In addition, the words soy, soya, and soja may have been used no later than 1700. Engelbert Kämpfer, a German naturalist and traveler who lived in Japan from 1690 to 1692, described the soybean and the manufacturing method of soy sauce (shoyu in Japanese) in his book “*Amoenitatum Exoticum*” with illustrations (Bowers, 1966; Kämpfer, 1712).

## Chemical structures and history of soy isoflavones

**Fig. 1:**
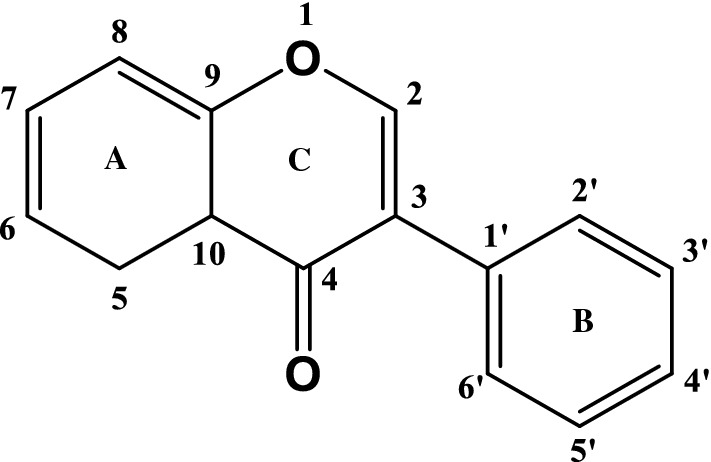
Structure and numbering of isoflavone skeleton

**Fig. 2:**
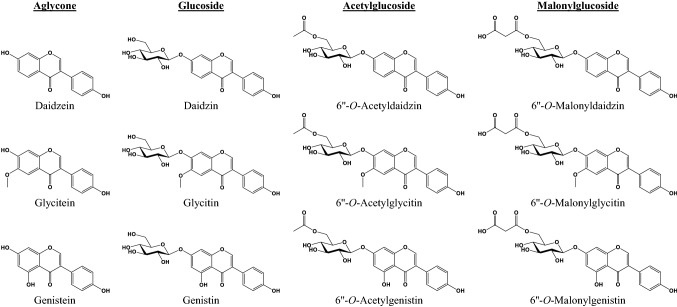
Chemical structures of isoflavone aglycones, glucosides, acetylglucosides, and malonylglucosides

Isoflavone is a subclass of flavonoids that has a diphenylpropane structure (C_6_–C_3_–C_6_) (Fig. 1) (Miadokova, 2009). The International Union of Pure and Applied Chemistry (IUPAC) nomenclature of the isoflavone backbone is 3-phenylchromen-4-one. The main structural difference between isoflavone and flavone is at which carbon of the C-ring the B-ring is placed in the flavonoid skeleton. The B-ring in isoflavone is attached to C-3, whereas that of the flavone is at C-2. Natural sources of isoflavones include the Fabaceae family, red clover (*Trifolium pratense*), alfalfa (*Medicago sativa*), kudzu (*Pueraria lobate*), and species of the genus *Genista* (Bustamante-Rangel et al., 2018). Among them, soybean is the major natural source for isoflavones. Soybeans mainly contain 12 kinds of isoflavones according to the type of aglycone and functional group (Fig. 2) (Popa and Rusu, 2017).

Three aglycones that determine the kind of soybean isoflavone are daidzein, genistein, and glycitein, according to the position and number attached to the hydroxyl (–OH) and methoxy (–OCH_3_) groups (Fig. 2). The IUPAC naming of daidzein is 7-hydroxy-3-(4-hydroxyphenyl)chromen-4-one. Structurally, two hydroxyl groups in daidzein are bonded to both ends (positions C-7 and C-4′) of the isoflavone core. The history and etymology of daidzein are believed to have come from *daidzu*, an old name for soybean in Japan (Kämpfer, 1712; Walz, 1931). Genistein (5,7-dihydroxy-3-(4-hydroxyphenyl)chromen-4-one) was first isolated from the dyer’s broom (*Genista tinctoria*) in 1899, and its structure was identified in 1926 (Baker and Robinson, 1926; Perkin and Newbury, 1899). Compared to daidzein, in genistein, one more hydroxyl group is bonded to the C-5 position. Later studies of soy isoflavones revealed that genistein and its derivatives were most abundant in soybeans (Walter, 1941; Walz, 1931). Glycitein (7-hydroxy-3-(4-hydroxyphenyl)-6-methoxychromen-4-one), a similar chemical name to the scientific name *Glycine*, was first isolated from soybean and named in 1973 (Naim et al., 1973). Unlike daidzein and genistein, glycitein is characterized by a unique methoxy group linked to the C-6 position of the isoflavone core (Ko, 2014). Compared to other soy isoflavones, glycitein and its derivatives have the lowest percentages in soybeans (Azam et al., 2020; Lee et al., 2015; Yatsu et al., 2016).

Soy isoflavone aglycones have common hydroxyl groups in positions C-7 and C-4′. Soy isoflavones are classified into 12 isoflavones according to the type of functional groups (glucose, acetylglucose (acetylated glucose), and malonylglucose (malonylated glucose) moieties) that bind to position C-7. Aglycones of soy isoflavones and their glycosides were isolated and characterized through hydrolysis using an acidic solvent (Walter, 1941; Walz, 1931). Among soy isoflavones combined to the acetylglucose group (as 6″-*O*-acetyl-7-*O*-*β*-d-glucoside), acetyldaidzin and acetylgenistin were first reported in 1979 and 1980, respectively, about half a century after the discovery of aglycones and non-acylated glucosyl isoflavones (daidzin, genistein, and glycitein) (Ohta et al., 1979; 1980). Theoretically, if the same biosynthetic pathway as acetylation of daidzin and genistin is applied to glycitin, acetylglycitin should also be present in soybeans. Nevertheless, no acetylglycitin other than daidzin and genistin with acetyl moiety was found (Ohta et al., 1979; 1980). Eventually, acetylglycitin was found about 10 years after the existence of acetyldaidzin and acetylgenistin (soy isoflavones with acetyl group) was reported. In 1991, the isolation and identification of acetylglycitin from soybean were achieved based on Ohta and her colleagues’ research (Kudou et al., 1991b). Furthermore, a total of nine isoflavones in soybeans were analyzed simultaneously using high-performance liquid chromatography (HPLC) (Kudou et al., 1991a).

The finding of malonyl isoflavones in soybean began with a change in extraction temperature. Kudou et al. (1991a), who discovered acetylglycitin, found that the isoflavone composition of soybean extracted with 70% aqueous ethanol at room temperature differed significantly from that of soybean extracted at 80 °C. This change in extraction method became the key to finding the three soy isoflavone glycosides bound to malonyl groups (6″-*O*-malonyl-7-*O*-*β*-d-glucoside) at a lower temperature, because the malonyl group decomposes at high temperatures (Mathias et al., 2006). Soy isoflavones, now up to 12 kinds, were first reported in 1991. Kudou et al. (1991a) were the first to present the composition ratios of 12 soy isoflavones, of which malonyldaidzin and malonylgenistin accounted for the largest portion of 66%. This is consistent with other reports saying that malonylglucosides of isoflavones are predominantly contained in foods such as soybeans and soy-based products (Azam et al., 2020; Bustamante-Rangel et al., 2018; Cho et al., 2020).

## Analytical methods for soy isoflavones

### Separation

Identification and quantification of isoflavones in soybean require a series of separating and detecting processes. Many analytical procedures have involved chromatographic methods, such as LC, thin layer chromatography (TLC), and gas chromatography (GC). These have played an important role in separating molecules ever since chromatography was first developed by Mikhail Semyonovich Tsvet in 1903 (Bustamante-Rangel et al., 2018; Wiley and Mclaren, 1955).

Coloration and extraction using silica-based TLC had been mainly used for separation and identification of compounds until the mid-1900s, before instrumental methods began to be commonly employed (Poole, 2003). Through TLC analysis, glycitein was first discovered from soybean in 1973 (Naim et al., 1973; 1974). Today, a high-performance TLC has been introduced, but it is less appropriate than other instrumental methods (GC and LC) when handling many samples through automated procedures (Puri and Panda, 2015).

The GC method has the same advantages of high resolution, high selectivity, and high sensitivity as the LC method (Bustamante-Rangel et al., 2018; Wang et al., 2002). However, the low volatility characteristics of isoflavone molecules require a derivatization process if being analyzed by GC. Therefore, due to the inherent characteristics of GC, such as the volatilization of samples and high column oven temperatures, the thermal stability of the soy isoflavones should be considered in order for proper analysis. For these reasons, when analyzing soy isoflavones using GC, complex biological samples such as urine, blood, and feces containing soy isoflavones are utilized rather than soybean and soy products (Wang et al., 2002).

Since the 12 kinds of soybean isoflavones have been established, the most widely used and prominent technique to separate and detect isoflavones is liquid phase-based chromatography (Kudou et al., 1991a). An LC system has many advantages such as less labor-intensive sample preparation, possible sample recovery, wide applicability, and reliable automated procedures (Bustamante-Rangel et al., 2018; Wahajuddin and Arora, 2013). In particular, LC-based analytical systems such as HPLC and ultra-performance liquid chromatography (UPLC) have been used successfully for the simultaneous analysis of soy isoflavones (Bustamante-Rangel et al., 2018). Thus far, many analytical techniques for detecting isoflavones in soybean and its associated foods using HPLC and UPLC have been proposed and have already been summarized and reviewed with a large number of references (Wahajuddin and Arora, 2013).

As technologies and instruments evolve, advanced separation techniques are constantly introduced. As a result, when referring to the LC method today, it generally embraces the meaning of “high-performance” or “ultra-high performance”. The word “performance” implies multiple meanings such as short analysis time, low detection limit, high resolution, and high reproducibility. When using the advanced LC method, the composition of the mobile phase, pH condition of the mobile phase, column temperatures, and column specifications should be considered (Wahajuddin and Arora, 2013).

In HPLC, reversed-phase (RP) separation assisted with octadecylsilyl silica (ODS) is the most common analytical technique for soy isoflavones. The two most common solvents, methanol and acetonitrile, are used as mobile phases in the LC separation system with RP columns. Methanol is a polar-protic solvent, whereas acetonitrile is a polar-aprotic solvent possessing a stronger dipole moment. Methanol is cheaper and less toxic than acetonitrile. Despite this, acetonitrile has a higher elution strength, faster retention time, and lower viscosity than methanol for PR-LC. Therefore, acetonitrile allows a short analysis time and high performance (Jang et al., 2019; Welch et al., 2009).

The pH of the mobile phase is one of the major factors for separation in an HPLC system with a packed column called an ODS column (Rosés et al., 1996). For the separation of ionizable compounds by the ODS column, an optimal pH of the mobile phase must be established (Bosch et al., 1996; Rosés et al., 1996). The theoretical retention factor of ionizable compounds at a certain mobile phase pH was first proposed by Horváth et al. (1977a). The retention factor depends on the acidity constant *K*_a_ of the acid–base equilibrium and the mobile phase pH (Gagliardi et al., 2006; Horváth et al., 1977a). Buffers are widely used to adjust the pH in HPLC systems (Bosch et al., 1996; Gagliardi et al., 2006; Rosés et al., 1996). There are many researchers that have proven that the adjustment of pH by adding buffers to the mobile phase affects the efficiency of the analysis (Bosch et al., 1996; Dorsey et al., 1998; Gagliardi et al., 2006; Rosés et al., 1996). Acidic buffers adjusted with trifluoroacetic acid, formic acid, acetic acid, and phosphoric acid are commonly used for soy isoflavone analysis (Bustamante-Rangel et al., 2018; Wahajuddin and Arora, 2013; Welch et al., 2009). Among these acidic modifiers, the acetic acid and formic acid, which are the simplest and most ubiquitous carboxylic acids with low molecular weights, provide a suitable pH range (pH 3–4) for the analysis of soy isoflavones (Khare et al., 1999; Lee et al., 2013; Welch et al., 2009). In contrast, the trifluoroacetic acid has a high acidity and volatility, and the phosphoric acid has a high viscosity such that they cannot be used in LC systems using mass detectors.

Temperature is the most important factor affecting the enthalpy in the separation process. In the 1970s, Horváth published a series of fundamental papers on the important influence of temperature in the RP-LC column (Horváth et al., 1977a; Horvath et al., 1977b). Analysis using ODS analytical columns is affected by ambient temperature differences changing with time throughout the day and with season throughout the year. According to Horváth’s theorem, columns exposed to different temperatures have different retention coefficients (Horváth et al., 1977a). Thus, temperature affects the reproducibility of the results (Bosch et al., 1996; Horváth et al., 1977a; Rosés et al., 1996). In general, higher column oven temperatures improve separation and reduce analysis time but restrict the column lifetime and stability of analyzed compounds such as isoflavone glucosides (Mathias et al., 2006). Simultaneous analyses of the flavone and flavonol isomers showed good separation when the temperature of the column oven was 40 °C rather than 20 °C (Jang et al., 2019). Isoflavones, however, had the highest resolution at 25 °C rather than 40 °C (Baranowska and Magiera, 2011). By using the HPLC equipped with an ODS column, when the column oven was set at 25 °C, 12 soy isoflavones were separated and simultaneously analyzed to quantifiable levels within 24 min (Cho et al., 2020).

According to recent trends, HPLC analysis uses high-efficiency columns, which involve short column lengths, small sized particles, and low flow rates. The simultaneous HPLC analysis of flavonoids with similar molecular structures and polarities, like different kinds of isoflavones, requires a high theoretical plate number (plate number ∝ column length/particle size) using long columns (25 cm or more). A longer column length provides higher resolution but entails a longer analysis time. Therefore, the ODS column, which utilizes a smaller particle size for higher resolution efficiency of the surface area rather than increasing the column length, has been developed. The smaller particle size leads to smaller pores in between particles through which the mobile phase can flow. This increases the pressure within the column, requiring the HPLC system and column to be resistant to high pressure to ensure their durability (Bustamante-Rangel et al., 2018). To avoid problems caused by high pressure applied to the HPLC system, many methods using short column lengths and low flow rates have been developed (Kiss et al., 2012; Vacek et al., 2008; Wahajuddin and Arora, 2013).

A UPLC system, which is run at higher pressures than HPLC, is typically equipped with a column that has a small particle size (less than 2.0 μm) and short column length (50–150 mm) (Raju et al., 2015). Columns with particles of small sizes generate high back pressure but achieve a high theoretical plate number, and therefore a high resolution. Moreover, the analytical efficiency of the UPLC system has been maximized through its combined usage with MS detectors and the technological development of the UPLC instrument. In particular, an increase in the amount of the mobile phase in the MS detector means that the inflow of the matrices increases. Therefore, the high-efficiency column fostered not only the high analytical efficiency but also the optimization of the analytical conditions (Griffiths, 2008).

Capillary electrophoresis (CE), one of the non-chromatographic separation techniques, uses the differences in the electrophoretic mobility of charged compounds on an electric field in small-diameter capillaries (50–100 μm I.D.) (Wang et al., 2002). The CE has inherent advantages such as high-resolution separation, the need for only small amounts of analytical solvent and sample, and short time of analysis (Bustamante-Rangel et al., 2018; Xiao et al., 2015). The analyses using CE techniques, such as capillary zone electrophoresis and micellar electrokinetic capillary chromatography, are generally performed based on a buffer run at alkaline pH. In general, the higher buffer concentrations and pH conditions in the CE technique require a longer run time (Mcleod and Shepherd, 2000; Xiao et al., 2015; Yatsu et al., 2016). In addition, the conjugated soy isoflavones are known to be unstable at high pH conditions (Bacaloni et al., 2005; Mathias et al., 2006). Therefore, the CE method limits the stability of the soy isoflavones, resolution, and analysis time (Mcleod and Shepherd, 2000; Xiao et al., 2015). These drawbacks may lead the CE technique to be less widely used for the analysis of isoflavones in foods than other separation techniques (Bustamante-Rangel et al., 2018; Mota et al., 2008).

### Detection

The first step for an analysis is separation through chromatography, followed by spectroscopy. Spectroscopy is the study of the radiation-matter interaction and deals with phenomena such as absorption, emission, diffraction, and fluorescence. For the analysis of soy isoflavones based on structural difference, the most important technique is spectroscopy. In the increasing order of wavelengths, ultraviolet (UV)-visible, infrared (IR), and radio waves are the spectral regions used in the most common forms of spectroscopy, such as UV–visible, IR, and NMR spectroscopy, respectively (Foudah and Abdel-Kader, 2017; Popa and Rusu, 2017). MS, which was first used by J. J. Thomson, is a more powerful technology than UV and IR spectroscopy (Thomson, 1897; Yates, 2011). Spectroscopic methods can obtain unique patterns of spectra of organic compounds and samples. Based on this feature, spectroscopic methods can be used to speculate the tentative structures of purified compounds after chromatographic separation.

IR spectroscopy was first discovered and used by William Herschel, famous for the discovery of Uranus (the seventh planet from the sun) in 1800 (Herschel, 1832). Herschel developed prism-based techniques for measuring IR spectra (Herschel, 1832; Rogalski, 2012). Later, the Fourier-transform IR spectroscopy that uses the interferometer was established as an analytical tool by Michelson (Michelson and Stratton, 1898). The IR spectroscopy uses vibrations that can be applied to almost all molecules that possess covalent bonds. Most molecules exhibit IR absorption in the middle IR region of 4000 to 400 cm^−1^ (2.5–25.0 mm wavelength) (Blum and John, 2011). The IR spectrum of soy isoflavones showed absorption bands for hydroxyl (3365 cm^−1^) and conjugated carbonyl (1694 cm^−1^) functional groups (Boonyaketgoson et al., 2015). On the contrary, it has been reported that the IR spectra of soy isoflavones were obtained in the wavenumber range of 2000–600 cm^−1^ (Iizuka and Aishima, 1999; Jose et al., 1974; Krähmer et al., 2013; Mulsow et al., 2015). The IR spectroscopy of soy isoflavones revealed that the quantitative values of pretreated samples were very similar to the results of the HPLC (Mulsow et al., 2015). In the case of foods, however, the pre-treatment of the sample or the post-processing of the data is necessary for IR spectroscopy because of the water (Iizuka and Aishima, 1999). Therefore, IR spectroscopy has been discussed as a suitable method for the rough analysis (finger printing) of soy isoflavones by using multivariate data, rather than quantitatively accurate analysis using purified compounds and samples of soy isoflavones (Wang et al., 2002).

Newton, the founder of classical mechanics, split sunlight using glass prisms in the seventeenth century (Newton, 1672). Coming to the twentieth century, numerous scientists such as Ångström, Beer, and Einstein eventually established light as quantum mechanics based on further research (Thomas, 1991). UV, which means beyond violet, is subdivided into UV-A (320–400 nm), UV-B (280–320 nm), and UV-C (200–280 nm) (Maverakis et al., 2010). UV spectroscopy provides the specific information of molecules based on quantum mechanics. When molecules absorb UV radiation, their electrons that make up the π bond or conjugation form (chromophore) are excited. Most of the flavonoid scaffolds have two main UV absorption spectra: where band I (300–380 nm) is associated with the cinnamoyl system (B-ring) and where band II (240–280 nm) is associated with the benzoyl system (A-ring) in flavonoids (Mabry et al., 1970a).

**Fig. 3:**
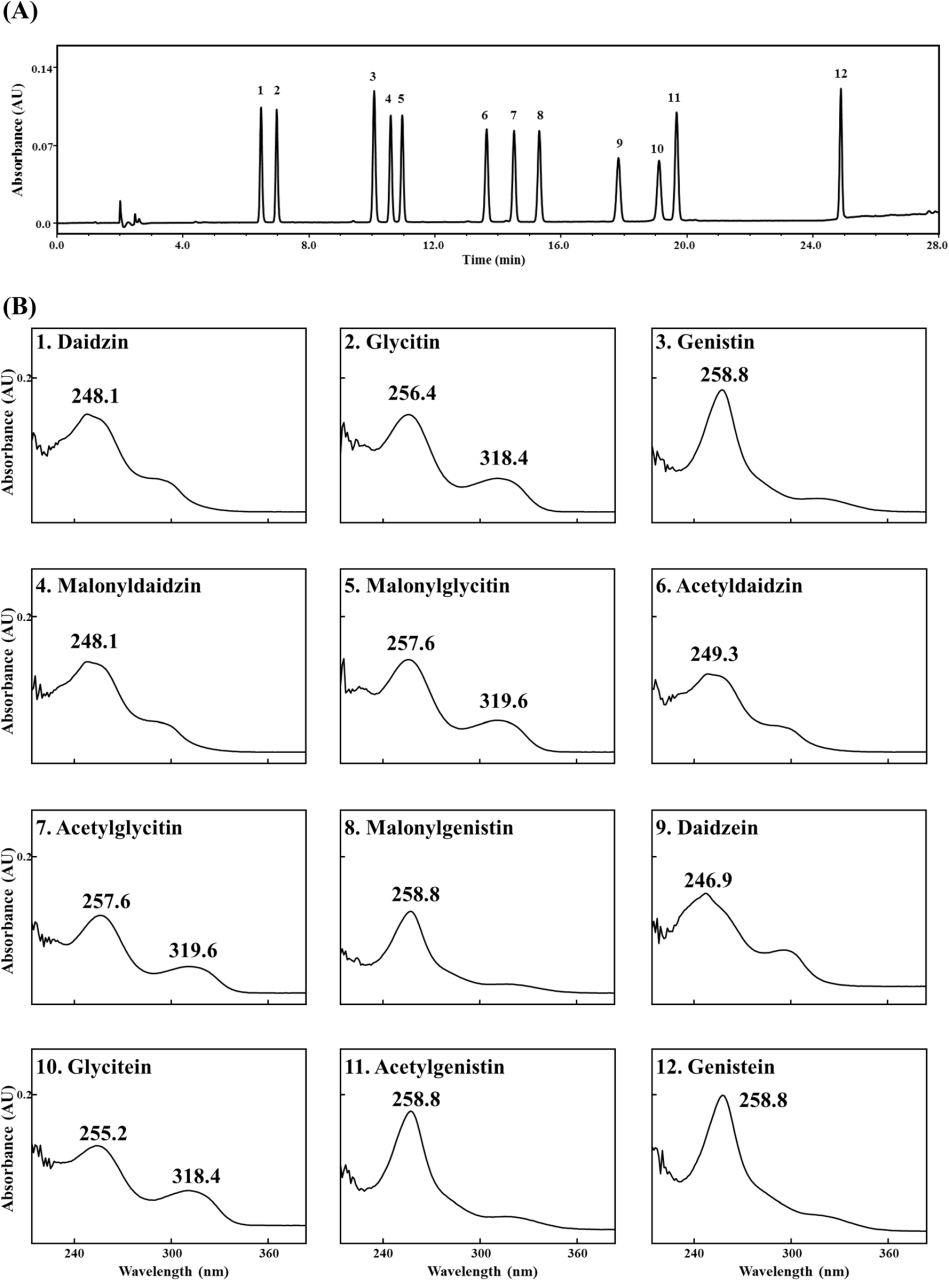
HPLC traces of simultaneous analysis of 12 soy isoflavone standards at 254 nm (**A**) and UV spectra at 200–400 nm (**B**). HPLC analytical conditions refer to Cho et al. (2020). Each isoflavone was used at a concentration of 100 μM

There are many reports that ascertained the soy isoflavones’ characteristic UV spectra (Foudah and Abdel-Kader, 2017; Mabry et al., 1970b). The UV absorption peak of soy isoflavone is at 245 to 270 nm. The shoulder peak of soy isoflavone is at 310 to 330 nm, because the B-ring attached to C-3 in the isoflavone affects the UV spectrum (Bustamante-Rangel et al., 2018; Foudah and Abdel-Kader, 2017). Figure 3 shows the elution order and UV spectra of 12 soy isoflavone standards analyzed using an HPLC coupled with a UV detector and RP column. The elution order of the 12 isoflavones is as follows in increasing retention time order: daidzin > glycitin > genistin > malonyldaidzin > malonylglycitin > acetyldaidzin > acetylglycitin > malonylgenistin > daidzein > glycitein > acetylgenistin > genistein (Fig. 3A). Isoflavone aglycones can be conjugated with three functional moieties, glucose, acetylglucose, and malonylglucose. Thus, isoflavone glucosides with the same aglycone have similar UV spectra (Fig. 3B). However, this similarity among soy isoflavones in UV spectra results in disadvantages during identification. Such disadvantages are increased when UV spectra are redshifted or blueshifted by analytical conditions. For example, metal ions or matrices in the sample induce redshift or blueshift in the UV spectrum pattern (Ducrey et al., 1995; Foudah and Abdel-Kader, 2017). To overcome the shortcomings (similar UV spectra and shifts) of UV spectroscopy, a way to increase peak resolution or to use high concentrations of analytes in chromatography is needed. In order to supplement the disadvantages such as difficulty of identification using UV detectors in UV spectroscopy, researchers began to use an MS detector (Bustamante-Rangel et al., 2018; Raju et al., 2015).

The importance of MS is represented by the fact that five pioneers in MS have been awarded the Nobel Prize. Among them, the first MS-related Nobel laureate J. J. Thomson studied the question “What is the nature of cathode rays?” which many scientists want to answer (Yates, 2011). On the question of whether the electron is a particle or a wave, Thomson measured the mass of the electron to prove the particle theory (Thomson, 1897). In the twentieth century, MS can measure the mass-to-charge ratio (*m*/*z*) of ions through sequential processes of the ionization, separation, and detection of molecules (Yates, 2011). Although innovative instruments such as UV and IR had appeared, MS was the sole instrument for measuring molecular mass until the 1950s. Klaus Biemann began his efforts to identify molecular structures in 1954 (Griffiths, 2008; Yates, 2011). The identification of molecular structures by MS raised the importance of molecular fragments. Then appeared the tandem MS (a.k.a. MS/MS or MS^2^) system capable of providing information about the masses of fragmented ions. The tandem MS, which connects two or more mass detectors, is a special approach compared to other spectroscopic methods such as UV, IR, and NMR. The tandem MS system can minimize the matrix effect and detect accurate molecular weight by connecting the same detectors in series. Since then, as electrospray ionization (ESI), matrix-assisted laser desorption ionization, and time of flight technologies have been developed, MS has arisen as the most powerful analytical tool (Fenn et al., 1989; Karas and Hillenkamp, 1988; Wiley and Mclaren, 1955).

Soy isoflavones are mainly analyzed using HPLC or UPLC coupled with single MS and tandem MS (Bustamante-Rangel et al., 2018; Smith and Udseth, 1988; Wahajuddin and Arora, 2013). The high sensitivity of MS is suitable for UPLC system conditions such as low sample volume and low volumetric flow of the mobile phase, which both can consequently increase the efficiency of analysis. Various MS methods have been developed for the analysis of soy isoflavones; atmospheric pressure chemical ionization (APCI) and ESI are commonly used among those (Kiss et al., 2012; Popa and Rusu, 2017). APCI is an ionization method mainly used for relatively less polar or nonpolar compounds of which acid–base reactions are involved in the gas phase (Rybak et al., 2008). On the other hand, ESI ionization is more suitable for polar compounds that can be ionized in solutions (McMaster, 2005). As polar compounds, isoflavones exist as ionized forms in the analyzing solution ([M − H]^−^ in negative mode or [M + H]^+^ in positive mode) (Popa and Rusu, 2017). Therefore, the ionizer of an MS system coupled with an LC uses ESI more often than APCI (Bacaloni et al., 2005; Popa and Rusu, 2017). The approach using MS is a very effective method to analyze soy isoflavones because soy isoflavones have different molecular weights. The calculated molecular weight of each soy isoflavone is shown in Table 1. The analysis of soy isoflavones using tandem MS has been reported in detail with fragment ions and fragmentation patterns under positive and negative ionization modes (Kang et al., 2007; Otieno et al., 2007; Raju et al., 2015; Ren et al., 2017).

**Table 1:** Chemical information and fragmentation patterns of individual soy isoflavones analyzed by mass spectrometry under negative and positive ion modes

Compound	Molecular formula	Calculated molecular weight	Abundance (%)	Major fragments (*m/z*) in negative mode		Major fragments (*m/z*) in positive mode	
				Raju et al. (2015)	Kang et al. (2007)	Andres et al. (2015)	Lee et al. (2015)
Daidzein	C_15_H_10_O_4_	254.0579	83.82	117, 133, 197	180, 195, 208, 223	65, 91, 137	
Glycitein	C_16_H_12_O_5_	284.0685	82.68	117, 163	184, 196, 211, 240, 268	118, 242, 270	
Genistein	C_15_H_10_O_5_	270.0528	83.62	107, 133, 163	159, 180, 201, 224, 240	65, 91, 153	
Daidzin	C_21_H_20_O_9_	416.1107	77.42	253		91, 199, 255	255
Glycitin	C_22_H_22_O_10_	446.1213	76.37	283		242, 270, 285	285
Genistin	C_21_H_20_O_10_	432.1056	77.24	269		91, 153, 271	271
Acetyldaidzin	C_23_H_22_O_10_	458.1213	75.53	253			255
Acetylglycitin	C_24_H_24_O_11_	488.1319	74.51	283			285
Acetylgenistin	C_23_H_22_O_11_	474.1162	75.35	269			271
Malonyldaidzin	C_24_H_22_O_12_	502.1111	74.34	253			255
Malonylglycitin	C_25_H_24_O_13_	532.1217	73.33	283			285
Malonylgenistin	C_24_H_22_O_13_	518.1060	74.16	269			271

The fact that flavonoids have common fragmentation patterns as a result of homogeneous bond cleavage is also applicable to the MS/MS analysis of soy isoflavones (Sawada et al., 2012). Soy isoflavones have various molecular weights of aglycones, such as those of daidzein (254 *m/z*), glycitein (284 *m/z*), and genistein (270 *m/z*), plus functional groups such as glucosyl (162 *m/z*), acetylglucosyl (204 *m/z*), and malonylglucosyl (248 *m/z*) (Pinheiro and Justino, 2012). Therefore, the fragmentation patterns of conjugated soy isoflavones are similar to one another because the same functional groups are detached from the isoflavone cores. Aglycone forms of soy isoflavones have the same value (119 *m/z*) of B-ring fragment ions from the C-ring cleavage through the retro-Diels–Alder fragmentation reaction (Maul et al., 2008). Fragments split from A-rings in the isoflavone aglycones daidzein, glycitein, and genistein have 137, 167, and 153 *m/z* values, respectively, under positive ion mode depending on the aglycone type (Kang et al., 2007; Maul et al., 2008; Otieno et al., 2007; Vukics and Guttman, 2010). In addition, soy isoflavone aglycones provide the characteristic fragment ions in the negative mode of the MS analysis. The neutral losses of CO, CO_2_, C_3_O_2_, and C_2_H_2_O from isoflavones take place prominently in the tandem MS analysis. The loss of CO_2_ is usually eliminated from the same ring (A- or C-ring) in the fragmentation of isoflavones (Kang et al., 2007). For example, genistein shows a fragmentation pattern that loses CO, CO_2_, C_3_O_2_, and H. Fragment ions observed in daidzein result from the loss of CHO, CO_2_ and H. On the other hand, glycitein has fragment ions that lose CO and H molecules after the special functional group CH_3_ is released. The major fragment ion values of soy isoflavones are shown in Table 1.

The origin of NMR began in 1924 with Wolfgang Pauli, a pioneer of quantum physics. His theory postulated a new degree of freedom for subatomic particles. Based on the concepts of the spin and magnetic moment of the electron, Felix Bloch and Edward M. Purcell, who shared the Nobel Prize in physics in 1952, first analyzed water and paraffin, respectively, using NMR in 1945 (Becker, 1993; Bloch, 1946; Kumar and Bhat, 2015; Pauli, 1940). Both having independently discovered NMR, Purcell and Bloch opened the road to new insights into the micro-world of nuclear physics according to Harald Cramér, member of the Royal Academy of Sciences (Kumar and Bhat, 2015). Over half a century, NMR has evolved further with the development of technologies such as the Fourier transform spectroscopy, invented by Richard R. Ernst and Weston A. Anderson in 1964, and the two-dimensional (2D) technique, invented by Richard R. Ernst in 1974 (Becker, 1993; Ernst, 1992). NMR spectroscopy uses lower energy radio frequency radiation (ν ≈ 1 to 10^3^ MHz) compared to other spectroscopic techniques (Bryce and Wasylishen, 2003). The basic rule of handling the NMR is to measure the spectrum of the behavior of atoms placed in a static external magnetic field (Moolenaar et al., 2003). The main spectral parameters consist of the chemical shift, spin–spin coupling, and signal intensity.

The two most basic NMR methods are the ^1^H- and the ^13^C-NMR. The ^1^H-NMR establishes the number of glycosides, anomeric configuration of glycosides, and the presence of specific functional groups based on information obtained from the resonant hydrogen atoms. The ^13^C-NMR is capable of determining the total number of carbons in the molecular backbone and the positions at which sugars are bonded through the resonant carbon atoms. The ^13^C-NMR is less sensitive than the ^1^H-NMR because ^13^C isotope exists in very small amounts of carbon isotopes and has a lower magnetogyric ratio ^1^H isotope. Therefore, a larger amount of samples and number of scans are normally required in ^13^C-NMR spectroscopy than in ^1^H-NMR spectroscopy (Günther, 2013). Similar to the tandem MS mentioned above, NMR scans are used in combination with ^13^C-NMR and ^1^H-NMR spectra. 2D NMR determines the position of an atom by detecting a correlation between different nuclei in the molecules. Some examples of the various types of 2D NMR are ^1^H-^1^H-correlation spectroscopy, heteronuclear multiple-bond correlation, heteronuclear single-quantum correlation, and distortionless enhancement by polarization transfer (Günther, 2013).

The discovery of the soy isoflavones mentioned above has always involved structural identification using NMR. Therefore, NMR data of soy isoflavones and their interpretation have been well reported (Jha et al., 1980; Sordon et al., 2017; Sung et al., 2004). Table 2 shows the NMR spectra of the soy isoflavone aglycones daidzein, glycitein, and genistein. These three isoflavone aglycones have different ^1^H-NMR spectra from one another due to the presence of hydroxyl or methoxy groups. Daidzein has a total of four ^1^H-NMR spectra at positions C-2, C-5, C-6, and C-8 on the chromane ring (A- and C-rings of the isoflavone core) among the three soy isoflavone aglycones. Glycitein, a methoxyisoflavone, is substituted by a methoxy group at position C-6 and a hydroxyl group at position C-7, and thus has a total of three ^1^H-NMR spectra (positions C-2, C-5, and C-8). Genistein shows a total of three ^1^H-NMR spectra at positions C-2, C-6, and C-8, because of its hydroxyl groups at positions C-5 and C-7. All three soy isoflavone aglycones have similar ^1^H-NMR shift values for protons in the B-ring. Characteristically, the B-ring of isoflavone aglycones has a symmetrical structure due to the hydroxyl group at position C-4′. Therefore, the ^1^H-NMR spectra at positions C-2′ and C-6′ as well as C-3′ and C-5′ have the same or similar chemical shifts (*δ*_H_ in ppm) in isoflavone aglycones. The ^13^C-NMR of soy isoflavone aglycones shows a similar chemical shift (*δ*_C_ in ppm) in the B-ring due to its symmetrical structure. Glycitein has one more ^13^C-NMR value due to the methoxy group at position C-6 compared to the other isoflavone aglycones. In addition, functional groups such as glucosyl, acetylglucosyl, and malonylglucosyl bound at position C-7 of each soy isoflavone aglycone affect ^1^H-NMR and ^13^C-NMR chemical shift values (Jha et al., 1980; Sordon et al., 2017; Sung et al., 2004).

Despite a powerful analytical technique approaching the atomic level, NMR has the disadvantages of large sample requirements (about 1 mg), limits in analytical atomic types, poor sensitivity, slow throughput, and difficulty in analysis of mixtures (Günther, 2013). Nevertheless, NMR is widely used for the elucidation of chemical structures and the identification of biological molecules such as proteins. In addition, the statistical processing of NMR data is used in fields such as metabolomics.

**Table 2:** ^1^H-NMR (*δ*_H_ in ppm, *J* in Hz) and ^13^C-NMR (*δ*_C_ in ppm) chemical shifts of daidzein, glycitein, and genistein

Position	Daidzein^a^		Glycitein^b^		Genistein^c^	
	*δ*_H_	*δ*_C_	*δ*_H_	*δ*_C_	*δ*_H_	*δ*_C_
2	8.29, s	152.26	8.27, s	152.08	8.32, s	154.01
3		122.67		123.40		123.88
4		178.58		174.73		181.37
5	7.96, d, 8.7	127.19	7.43, s	104.89		163.70
6	6.93, d, 2.2	115.05		146.95	6.26, d, 2.1	99.73
7		162.62		152.96		164.73
8	6.85, d, 2.2	102.15	6.93, s	102.94	6.42, d, 2.1	94.34
9		157.64		152.08		158.82
10		116.87		116.57		106.05
11			3.88, s	55.88		
1′		123.91		122.91		122.90
2′	7.39, d, 8.5	129.98	7.39, d, 8.3	130.01	7.37, d, 8.4	130.97
3′	6.81, d, 8.5	115.05	6.81, d, 8.3	115.10	6.82, d, 8.4	115.82
4′		157.33		157.30		158.20
5′	6.81, d, 8.5	115.05	6.81, d, 8.3	115.10	6.82, d, 8.4	115.82
6′	7.39, d, 8.5	129.98	7.39, d, 8.3	130.01	7.37, d, 8.4	130.97

^a^H shift values derived from Sung et al. (2004) and Sordon et al. (2017), C shift values derived from Jha et al. (1980)

^b^H shift values derived from Sung et al. (2004), C shift values derived from Jha et al. (1980)

^c^H shift values derived from Sordon et al. (2017), C shift values derived from Jha et al. (1980)

In conclusion, soybean is one of the most interesting and important materials in our daily diet and food industry. This review has addressed the origins of soybeans and the history of isoflavone identification in chronological order based on many reports. Isoflavones, the major biologically active compounds in soybeans, have been discovered and reported to have 12 different types using various methods, such as LC and LC/MS. The spectroscopic approach to detecting and identifying isoflavones provides background knowledge for the analysis of isoflavones in soybeans and soy-derived foods. Among the spectroscopic methods of isoflavone analysis, LC coupled with UV and MS is the most powerful and efficient system. We look forward to see the preparation methods and the LC-UV/MS system advance in the future to follow up the food processing that is becoming more diversified and complex in our industry.
