# Correction to: A brief history and spectroscopic analysis of soy isoflavones

**DOI:** 10.1007/s10068-020-00845-0

**Published:** 2020-11-20

**Authors:** Young Sung Jung, Chan-Su Rha, Moo-Yeol Baik, Nam-In Baek, Dae-Ok Kim

**Affiliations:** 1 Department of Food Science and Biotechnology, Kyung Hee University, Yongin, 17104 Republic of Korea; 2 Department of Oriental Medicinal Biotechnology, Kyung Hee University, Yongin, 17104 Republic of Korea

## Correction to: Food Sci Biotechnol 10.1007/s10068-020-00815-6

The article “A brief history and spectroscopic analysis of soy isoflavones”, written by Young Sung Jung, Chan-Su Rha, Moo-Yeol Baik, Nam-In Baek, Dae-Ok Kim, was originally published electronically on the publisher’s internet portal on 15 September 2020 without open access. With the author(s)’ decision to opt for Open Choice the copyright of the article changed on 26 October 2020 to © The Author(s) 2020 and the article is forthwith distributed under a Creative Commons Attribution 4.0 International License, which permits use, sharing, adaptation, distribution and reproduction in any medium or format, as long as you give appropriate credit to the original author(s) and the source, provide a link to the Creative Commons license, and indicate if changes were made. The images or other third party material in this article are included in the article’s Creative Commons license, unless indicated otherwise in a credit line to the material. If material is not included in the article’s Creative Commons license and your intended use is not permitted by statutory regulation or exceeds the permitted use, you will need to obtain permission directly from the copyright holder. To view a copy of this license, visit http://creativecommons.org/licenses/by/4.0.

The original article has been corrected.

